# Synthesis, Characterization and Thermal Studies of Zn(II), Cd(II) and Hg(II) Complexes of *N*-Methyl-*N*-Phenyldithiocarbamate: The Single Crystal Structure of [(C_6_H_5_)(CH_3_)NCS_2_]_4_Hg_2_

**DOI:** 10.3390/ijms12031964

**Published:** 2011-03-17

**Authors:** Damian C. Onwudiwe, Peter A. Ajibade

**Affiliations:** Department of Chemistry, University of Fort Hare, Private Bag X1314, ALICE 5700, South Africa; E-Mail: donwudiwe@ufh.ac.za

**Keywords:** dithiocarbamate, thermal studies, group 12 complexes, crystal structure

## Abstract

Zn(II), Cd(II) and Hg(II) complexes of *N*-methyl-*N*-phenyl dithiocarbamate have been synthesized and characterized by elemental analysis and spectral studies (IR, ^1^H and ^13^C-NMR). The single crystal X-ray structure of the mercury complex revealed that the complex contains a Hg centre with a distorted tetrahedral coordination sphere in which the dinuclear Hg complex resides on a crystallographic inversion centre and each Hg atom is coordinated to four S atoms from the dithiocarbamate moiety. One dithiocarbamate ligand acts as chelating ligand while the other acts as chelating bridging ligand between two Hg atoms, resulting in a dinuclear eight-member ring. The course of the thermal degradation of the complexes has been investigated using thermogravimetric and differential thermal analyses techniques. Thermogravimetric analysis of the complexes show a single weight loss to give MS (M = Zn, Cd, Hg) indicating that they might be useful as single source precursors for the synthesis of MS nanoparticles and thin films.

## 1. Introduction

Dithiocarbamates are versatile ligands with a wide range of chemistry. The application of dithiocarbamate ligands have been demonstrated in the construction of new supramolecular structural motifs such as polymetallic nanosized macrocycles [1–5]. Group 12 metal complexes of dithiocarbamates continue to attract attention because of various industrial and biological applications [6–10]. These classes of inorganic compounds are large and some of their studies were carried out to understand the interactions which exist between the metal ions and the ligands [9]. In recent years, the preparation and investigation of well-defined nanocrystal of MS semiconductors, where M = Zn, Cd and Hg, have been the focus of considerable attention because of the ability to fine-tune their electronic and optical properties for possible applications [11–13]. The dithiocarbamate complexes are important sources of metal chalcogenides (MS) in solid-state materials. For instance, HgS has been found to demonstrate, amongst other phenomena, strong room-temperature infrared luminescence [14] and can thus be a good material for integration into light emitting devices such as semiconductor nanoparticles [15]. The metal complexes are also useful precursors for deposition of II/VI compound semiconductor materials because of their reasonable volatility and less carbon deposition as impurity [16]. In this study, we present the synthesis and characterization of Zn(II), Cd(II), and Hg(II) complexes of *N*-methyl-*N*-phenyl dithiocarbamate and their thermal studies to evaluate the potential of the complexes as single source precursors for the preparation of II–VI semiconductor nanoparticles.

## 2. Results and Discussion

### 2.1. Synthesis

The reaction of CS_2_ with a secondary aromatic amine in the presence of concentrated aqueous NaOH at about 0 °C lead to the formation of sodium *N*-methyl-*N*-phenyl dithiocarbamate [17]. The complexes were obtained as air stable compounds at room temperature by the reaction of the ligand with their respective metal salts in 2:1 mole ratio. Elemental analyses and spectroscopic studies agree with the proposed formulation for the complexes. The mechanisms for the formation of the ligands and complexes are as follows: 
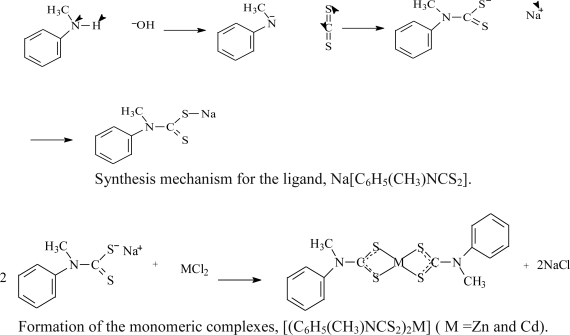
 
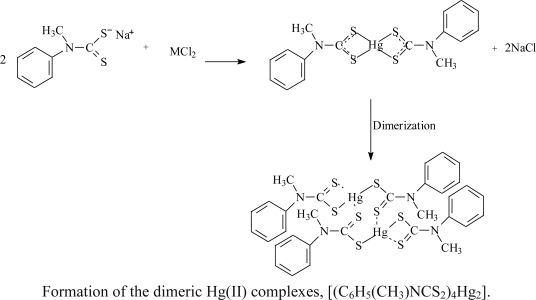


### 2.2. Spectroscopic Analysis

The IR spectra of the complexes and the ligand were compared and assigned on careful comparison. Three main regions are of interest in dithiocarbamate compounds: the 1580–1450 cm^−1^ region primarily associated with the stretching of the C—N of NCS_2_^−^; the 1060–940 cm^−1^ region, associated with ν(—CSS); and the 420–250 cm^−1^ region which is associated with ν(M—S) [18]. The strong bands at about 1450–1491 cm^−1^ in all the complexes are attributed to the ν(C—N) stretching vibration. This band is observed at a lower frequency in the free ligand (1430–1454 cm^−1^) and indicates an increase of the carbon-nitrogen double bond character, caused by electron delocalization toward the metal center upon coordination to the metal atoms [19]. It is found that the coordination mode of alkyl-aryl dithiocarbamate ligands with group 12 metals is bidentate by the sulfur atoms [20–22]. This is consistent with the crystal structure of the mercury complex. The ν(CS_2_)_asym_ and ν(CS_2_)_sym_ which appear at 1055 cm^−1^ and 961 cm^−1^ in the ligand [23] are replaced by strong singlet at about 1000 cm^−1^ in all the complexes indicating that the dithiocarbamate moiety is symmetrically coordinated to the metal ions [24]. It has been shown that the presence of only one band in the 1000 ± 70 cm^−1^ region is characteristic of a bidentate nature for the dithiocarbamate moiety, while the splitting of the same band within a difference of 20 cm^−1^ in the same region is due to the monodentate binding of dithiocarbamate ligand [25]. The ν(C—H) stretching for the methyl group is shown in the region 2925–2850 cm^−1^ while the C—H bending modes appeared as an intense band around 1356 cm^−1^ in all the compounds [26]. The ν(=C—H) stretching of the aromatic ring which occurs slightly above 3000 cm^−1^ [27] is observed between 3057 and 3080 cm^−1^ while σ(=C—H) bending modes of the aromatic ring occurred around 700 cm^−1^ [27,28]. The spectra of both the ligand and the complexes showed two bands in the region 1620–1550 cm^−1^ that may be assigned to ν (C=C) of the aromatic ring. The M—S vibration occurs at far infra red region.

The ^1^H NMR of the complexes contain a sharp singlet, corresponding to three protons, in the region 3.82–3.64 ppm, ascribed to methyl linked directly with N atoms contained in dithiocarbamate. A downfield by δ = 0.4–0.6 ppm as compared to the chemical shifts of dimethyl dithiocarbamate (observed in the range δ = 3.26–3.40) [27] is observed. The difference could be due to the effect of the electronegativity of nitrogen atom compared to alkyl carbon [29]. It is shown that the coordinated dithiocarbamate group is more electronegative than in the case where there is no coordination [23]. The multiple signals observed in the region δ = 7.53–7.40 ppm are attributed to the protons of phenyl rings. ^13^C NMR spectra of the complexes exhibit weak signals in the region 190.51–202.10 ppm assign to NCS_2_ carbon atoms of the dithiocarbamate moieties. Signals observed at 47.24, 45.40, and 48.78 ppm for the Zn, Cd and Hg complexes respectively correspond to methyl carbon attached to the nitrogen atom. The signals due to the carbons of aryl groups were exhibited between 147.17–126.31, 148.55–126.44, and 129.65–125.38 ppm in the Zn, Cd and Hg complexes respectively.

### 2.3. Thermal Analyses of the Complexes

**Figure 1.**
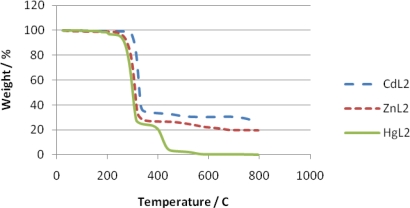
TGA curves showing the degradation of complexes.

**Figure 2.**
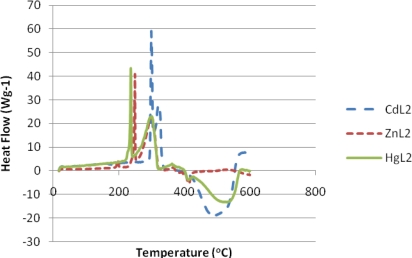
DSC curves of the complexes (in nitrogen) at a heating rate of 5 °C min^−1^.

**Figure 3.**
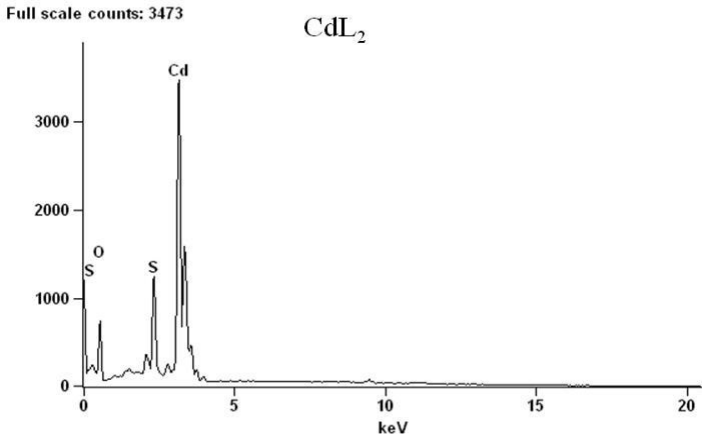
EDX of the decomposed products from complex CdL_2_ at 800 °C.

**Figure 4.**
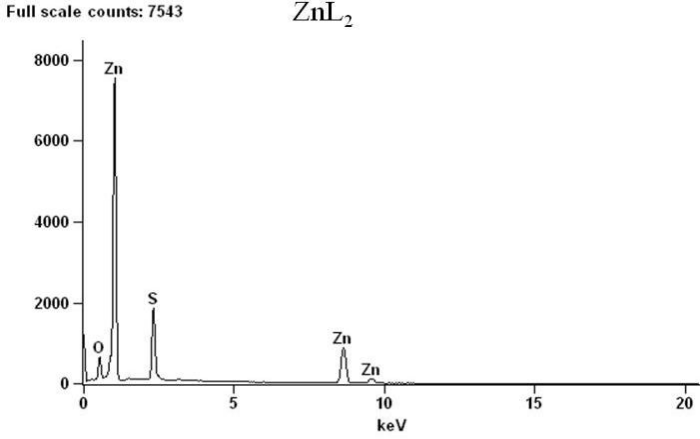
EDX of the decomposed products from complex ZnL_2_ at 800 °C.

The thermal properties of the complexes were studied by TGA and DSC in the temperature ranging from 20 to 800 °C under nitrogen atmosphere. The content of a particular component in a complex changes with its composition and structure. These can be determined based on mass losses of these components in the thermogravimetric plots of the complex. The pertinent thermal decomposition data for the complexes are presented in Table 1, Figure 1 shows their degradation pattern and the DSC curves of the complexes are presented in Figure 2. The compounds start decomposing above 210 °C and the thermogram for each complex exhibits two distinct decomposition steps at 219, 270, 168 and 450, 430, 361 °C for the Zn, Cd and Hg complexes respectively. The first decomposition step stretches beyond 50 °C and exhibits 65–70% weight loss. This corresponds to decomposition of the organic moiety [30] leaving behind metal sulfide as the end product. The slight weight loss (<2%) observed in the mercury complex around 185 °C could be ascribed to the presence of entrapped water or solvent molecule [31]. The absence of any thermal change before this temperature is reached indicates that samples restructuring did not take place before the degradation processes started [32], and also demonstrates their high thermal stability. The second decomposition temperature stretches to around 650 °C in Zn and Cd complexes but less than 600 °C in Hg complex. The products correspond to the respective metal oxides except in the Hg complex where the thermogram indicates volatilization). The presence of oxygen and sulfur in the end product of the zinc and cadmium complexes at 800 °C, as shown by the EDX result (Figures 3 and 4), may indicate oxysulfate which probably have formed due to the oxidation of the sulfide. It is evident from the thermogram (Figure 1) that the Hg complex has the least thermal stability as previously observed [33]. The calculated mass change agrees favorably with the experimental values. The anomaly observed in the mercury complex may be due to the volatility in the second phase of decomposition. The stability trend [34] of the complexes could be said to follow: Hg < Zn < Cd.

In the DSC curve, all the complexes show a sharp endothermic curve above 200 °C indicating their melting points. Comparing the TG and DSC curves, it is well visible that the mass loss process starts after the melting of the chelate, *i.e.*, in its liquid-state [35]. The broad exothermic hump observed in Cd and Hg complex imply slow decomposition leading to volatilization upon heating [31] as observed in the Hg complex thermogram.

### 2.4. Molecular Structure of the Mercury Complex

**Figure 5.**
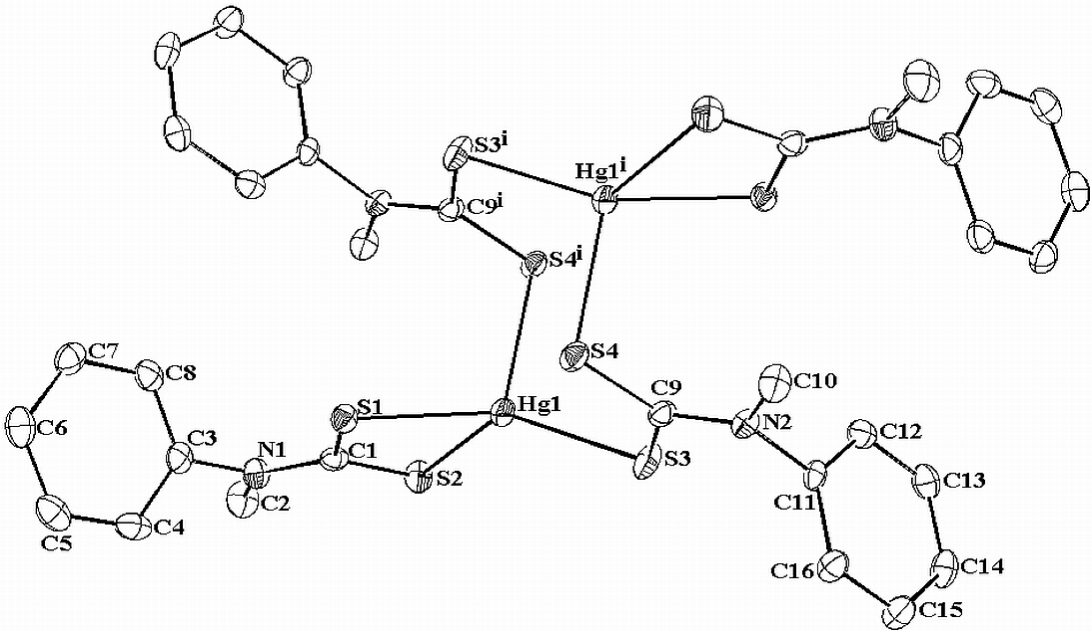
Molecular structure of [(C_6_H_5_)(CH_3_)NCS_2_]_4_Hg_2_. The thermal ellipsoids are shown at 50% probability level.

The mercury complex was structurally determined by X-ray crystallography. The crystallographic and measurement data are shown in Table 2 and representative bond lengths and angles are listed in Table 3. Figure 5 shows the thermal ellipsoid representations of the mercury complex.

The structure determination of the mercury complex, [(C_6_H_5_)(CH_3_)NCS_2_]_4_Hg_2_ at 100 (2)K has monoclinic (P21/c) symmetry. The complex contains Hg centre with a distorted tetrahedral coordination sphere in which the dinuclear Hg complex resides on a crystallographic inversion centre and each Hg atom is coordinated to four S atoms from the dithiocarbamate moiety. One dithiocarbamate ligand acts as chelating ligand while the other acts as chelating-bridging ligand between two Hg atoms resulting in dinuclear eight-member ring (defined by the atoms [Hg1-S3-C9-C4]_2_). There Hg-S distances are 2.4114 (9), 2.4810 (9), 2.6956 (9) and 2.7327 (9) Å). There is a short Hg…..Hg contact of 3.9297 (4) Å. A number of reports on the crystal structures of Hg(II) dithiocarbamates Have been made where they exist as both mononuclear [Hg(S_2_CNR_2_)_2_], and binuclear complexes [Hg_2_(S_2_CNR_2_)_4_] [36–39]. In mononuclear complexes, both dithiocarbamate ligands are coordinated in an S,S'-bidentate fashion by the mercury atom surrounded by four S atoms. However, the geometry of [HgS_4_] can be both tetrahedral [37,38]; and square planar [36,40].

In the present study, the structure of the mercury complex crystallizes with half a molecule [Hg{S_2_CN(C_6_H_5_)(CH_3_}_2_] in asymmetric unit, the other half is generated by an inversion symmetry through additional Hg-S bonds. The Hg…..Hg distance is 3.9297 (4) Å. The compound adopts a centrosymmetric structure in which the Hg atom in the mononuclear fragment [Hg{S_2_CN(C_6_H_5_)(CH_3_}_2_] coordinates fairly strongly to two S atoms of a dithiocarbamate ligand to form planar four-membered chelate ring defined by Hg-S2-C1-S1. This chelate ring is characterized by short Hg…C1 contacts (2.9825 Å) which is slightly longer than the longest Hg-S bond length (Hg1-S2, 2.7372 (9) Å). In addition, the S1…S2 distance is 2.994 Å which allows for a *trans*-annular effect in which Hg1 interacts directly to C1 through space in the four-membered chelate ring [39]. The binding of this dithiocarbamate ligand can be described as anisobidentate by virtue of one of the Hg-S bond distances being longer than the other (2.4810 (9) and 2.7327 (9) Å for Hg1-S1 and Hg1-S2, respectively). A second dithiocarbamate ligand has mixed structural function, chelating and bridging, and is involved in the dimerization of two monomeric fragments. The chelating Hg-S bond is stronger (Hg-S3, 2.4114 (9) Å) than the bridging Hg-S bond (Hg1-S4^i^, 2.6956 (9) Å; Symmetry operator for i = −*x* + 2, −y, −z + 1). The longest of the four Hg-S bond distances is however still smaller than the sum of *van der Waals* radii of Hg and S (3.3 Å) [41]. The dinuclear complex therefore has an eight-member cyclic core (Hg_2_S_4_C_2_) with an approximate chair conformation in which the atoms Hg1-S4-Hg-S4 form the base of the chair while S3 and C4 atoms lie out of the plane formed by this base in a plane that is almost perpendicular to it (torsion angles S4^i^-Hg1-S3-C9 = −88.22 (13)°; Symmetry operator for i = − *x* + 2, −y, −z + 1 and Hg1^i^-S4^i^-C9-S3 = 86.0 (2)°; Symmetry operator for i = − *x* + 2, −y, −z + 1), serving as part of the chair conformation.

Similar to other dithiocarbamates, the C_2_NC(S)S fragment is planar and the dihedral angle between the planes formed by this fragment from the two dithiocarbamate ligands in the mononuclear fragments is 22.60 (5)°. The N-C(S)S bond (1.347 (5) Å) is appreciably stronger than the N-CPh (1.444 (5) and the N-CCH3 (1.469 (5) Å bonds. The angles around the N atom are also close to 120° (Table 3) hence an admixture of the *sp^2^* state to *sp^3^* hybrid of the N atom [39]. The angles around the Hg atom are S3-Hg1-S1 = 146.42 (3), S3-Hg1-S4^i^ = 102.35 (3), S1-Hg1-S4^i^ = 107.67 (3), S1-Hg1-S2 = 69.91 (3), S4^i^-Hg1-S2 = 95.46 (3) and S3-Hg1-S2 = 122.15 (3)°; (Symmetry operator for i = − *x* + 2, −y, −z + 1) which at first approximation indicates a distorted tetrahedron geometry around the mercury atom. The trigonal base formed by the three chelating S atoms and the Hg atom show a distorted trigonal planar geometry in which the Hg atom is 0.5762 (2) Å out of this plane.

**Figure 6.**
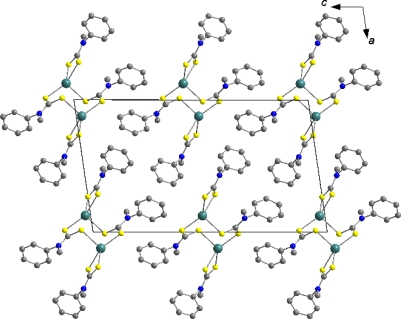
Packing diagram of [(C_6_H_5_)(CH_3_)NCS_2_]_4_Hg_2_ as viewed down the crystallographic *b* axis.

**Figure 7.**
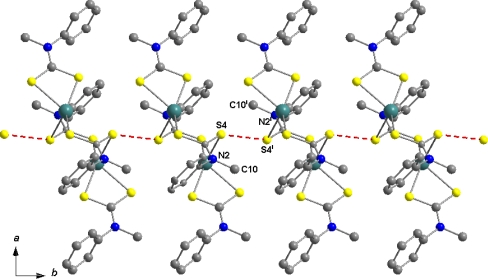

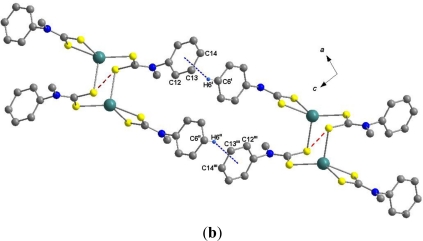
**(a)** Packing diagram of [(C_6_H_5_)(CH_3_)NCS_2_]_4_Hg2 showing S4···S4 intermolecular interactions; **(b)** Packing diagram of [(C_6_H_5_)(CH_3_)NCS_2_]_4_Hg_2_ showing C—H···π intermolecular interactions.

The packing of the complex in the unit cell (Figure 6) is characterized by S….S (Figure 7a) and C-H…л intermolecular interactions (Figure 7b). S….S intermolecular interactions (S4….S4 =3.560(*x*)Å), (symmetry operator = 2 – *x*, 1 – y, 1− z) link dinuclear molecules along the crystallographic b axis. The C—H…л intermolecular interactions H6….π = 2.9565 (2)° and <C6–H6….π = 155 (2)°; (symmetry operator = *x* + 1, −y + ½, z + ½) join S4….S4 also along the crystallographic b axis. The structural diversity observed in the Hg(S_2_CNR_2_)_2_ compounds can be ascribed to intra- or intermolecular association giving rise to distortions from linearity and result in varied coordination geometries [37,42]. The additional interactions observed in the title compound are dictated by the need to maximize intermolecular associations made possible by the absence of steric restrictions precluding association in the lattice. Hence, Hg-S interactions are present which give rise to dimeric structures.

## 3. Experimental Section

### 3.1. Materials and Methods

The ligand, sodium *N*-methyl-*N*-phenyldithiocarbamate (L) was prepared according to the method previously described in [17]. Other reagents and solvents employed were commercially available and used without further purification. Elemental analyses were performed using Fisons elemental analyzer. The FT-IR spectra (KBr pellets) were recorded using a Perkin Elmer 2000 FT-IR spectrometer in the range of 4000–370 cm^−1. 1^H and ^13^C NMR spectra were recorded on 400 MHz and 101 MHz Bruker NMR spectrophotometers respectively. Chemical shifts are given in ppm (δ scale) relative to tetramethylsilane (for ^1^H and ^13^C nuclei).

### 3.2. Synthesis of the Ligand: Sodium N-Methyl-N-phenyldithiocarbamate, Na[C_6_H_5_(CH_3_)NCS_2_]

The ligand was prepared by the addition of 12.1 mL (0.2 mol) of carbon disulphide (density 1.2) into an ice cold solution of sodium hydroxide (8 g, 0.2 mol) dissolved in 10 mL of distilled water. To the solution, 21.80 mL of *N*-methyl aniline (density 0.985) was added and the mixture was stirred for about 2 h while ensuring the temperature was less than 4 °C. A yellowish-white solid product separated out which was filtered, washed with small portion of ether. Pure [Na(C_6_H_5_(CH_3_)NCS_2_)] was obtained by recrystallization from acetone. Yield: 84%. Selected IR, (cm^−1^) **L**: 1454 υ(C=N), 1262 υ(C_2_—N), 961, 1055 υ(S=C=S), 3300 υ(O—H), 1624 σ(O—H).

### 3.3. Preparation of Complexes

The preparation of the complexes were carried out using the same experimental procedure as follows: 25 mL aqueous solution of the respective metal salts (1.25 mmol) [Zn(OOCCH_3_)_2_, CdCl_2_·½H_2_O, HgCl_2_], was added to 25 mL aqueous solution of sodium *N*-methyl-*N*-phenyldithiocarbamate (0.512 mg, 2.50 mmol). Solid precipitates formed immediately and the mixture was stirred for about 45 mins, filtered off and rinsed several times with distilled water and recrystallized with appropriate solvents.

#### 3.3.1. Bis-(*N*-methyl-*N*-phenyldithiocarbamato)zinc(II): **ZnL_2_** **(1)**

Complex was obtained as white solid. Yield: 0.438 g, (81.72 %), M.p. 246–248 °C.

^1^H NMR (DMSO) δ = 7.46 – 7.19 (m, 10H, –C_6_H_5_), 3.64 (s, 6H, –CH_3_).
^13^C NMR (DMSO) δ 147.17, 129.55, 127.81, 126.31(−C_6_H_5_), 47.24(−CH_3_), 190.51(−CS_2_).
Selected IR, υ (cm^−1^): 1491 (C=N), 1261 (C_2_-N), 969 (C=S). *Anal.* Calc. for C_16_H_16_N_2_S_4_Zn (429.94): C, 44.70; H, 3.75; N, 6.52; S, 29.83. Found: C, 44.69; H, 3.80; N, 6.90; S, 29.59.
The product was recrystallized in dichloromethane.

#### 3.3.2. Bis-(*N*-methyl-*N*-phenyldithiocarbamato)cadmium(II): **CdL_2_** **(2)**

Complex was obtained as white solid. Yield: 0.559 g (93.95 %), M.p. 296–298 °C.
^1^H NMR (DMSO) δ = 7.46–7.19 (m, 10H, −C_6_H_5_), 3.64 (s, 6H, −CH_3_).
^13^C NMR (DMSO) δ 148.55, 129.39, 128.79, 126.44 (−C_6_H_5_), 45.40 (−CH_3_), 202.10 (−CS_2_).
Selected IR, υ (cm^−1^): 1490 (C=N), 1255 (C_2_-N), 963 (C=S). *Anal.* Calc. for C_16_H_16_N_2_S_4_Cd (476.97): C, 40.29; H, 3.38; N, 5.87; S, 26.89. Found: C, 39.96; H, 3.36; N, 6.05; S, 27.31

#### 3.3.3. Bis(μ-*N*-methyl-*N*-phenyldithiocarbamato-S:S’)bis-[(*N*-methyl-*N*-henyldithiocarbamato) Mercury(II)]: **Hg_2_L_4_ (3)**

Complex was obtained as yellow solid. Yield: 0.600 g (84.51%), M.p. 235–236 °C.
^1^H NMR (CHCl_3_) δ = 7.53–7.29 (m, 20H, −C_6_H_5_), 3.82 (s, 12H, −CH_3_).
^13^C NMR (CHCl_3_) δ 129.65, 128.35, 125.38 (−C_6_H_5_), 48.78 (−CH_3_), 201.20 (−CS_2_).
Selected IR, υ (cm^−1^): 1491 (C=N), 1255 (C_2_-N), 959 (C=S). *Anal.* Calc. for C_32_H_32_N_4_S_8_Hg_2_ (565.15): C, 34.00; H, 2.85; N, 4.96; S, 22.69. Found: C, 34.02; H, 2.95; N, 5.04; S, 22.81.
The product was recrystallized from a dichloromethane/ethyl acetate (3:1 v/v) mixture to afford yellow crystals suitable for X-ray crystallography.

### 3.4. Thermal Studies

Thermogravimetric analyses experiments were carried out on a Perkin Elmer thermogravimetric analyzer (TGA 7) fitted with a thermal analysis controller (TAC 7/DX). Samples of 10–12 mg of each complex were loaded into an alumina cup and weight changes were recorded as a function of temperature for a 10 °C min^−1^ temperature gradient between 20 °C and 800 °C. A purge gas of flowing nitrogen at a rate of 20 mL min^−1^ was used. The differential-scanning calorimetry at high temperature was performed with a Thermo scientific DSC (i–series) instrument for temperatures ranging from 20 to 600 °C at a rate of 5 °C min^−1^, in nitrogen atmosphere.

### 3.5. Crystal Structure Determination

A yellow crystal with approximate dimensions 0.27 × 0.18 × 0.17 mm^3^ was selected under oil under ambient conditions and attached to the tip of a MiTeGen MicroMount©. The crystal was mounted in a stream of cold nitrogen at 100(2) K and centered in the X-ray beam by using a video camera. The crystal evaluation and data collection were performed on a Bruker SMART APEXII diffractometer with Cu Kα (λ = 1.54178 Å) radiation and the diffractometer to crystal distance of 4.03 cm. The initial cell constants were obtained from three series of w scans at different starting angles. Each series consisted of 35 frames collected at intervals of 0.7° in a 25° range about w with the exposure time of 3 seconds per frame. The reflections were successfully indexed by an automated indexing routine built in the APEXII program. The final cell constants were calculated from a set of 3206 strong reflections from the actual data collection. The data were collected by using the full sphere data collection routine to survey the reciprocal space to the extent of a full sphere to a resolution of 0.82 Å. A total of 27,791 data were harvested by collecting 19 sets of frames with 0.7° scans in w with an exposure time 4–8 sec per frame. These highly redundant datasets were corrected for Lorentz and polarization effects. The absorption correction was based on fitting a function to the empirical transmission surface as sampled by multiple equivalent measurements [43]. The systematic absences in the diffraction data were uniquely consistent for the space group *P*21/*c* that yielded chemically reasonable and computationally stable results of refinement [44]. A successful solution by the direct methods provided most non-hydrogen atoms from the *E*-map. The remaining non-hydrogen atoms were located in an alternating series of least-squares cycles and difference Fourier maps. All non-hydrogen atoms were refined with anisotropic displacement coefficients. All hydrogen atoms were included in the structure factor calculation at idealized positions and were allowed to ride on the neighboring atoms with relative isotropic displacement coefficients.

## 4. Conclusions

Zn(II), Cd(II) and Hg(II) complexes of *N*-methyl-*N*-phenyldithiocarbamate have been synthesized and characterized by elemental analyses and spectroscopic techniques. Four coordinate geometries are proposed for the Zn(II) and Cd(II) complexes. Single crystal X-ray structure of the Hg(II) complex revealed that the complex is dimeric and the coordination geometry around each mercury atom is a distorted tetrahedral. Thermogravimetric analysis of the complexes showed a single weight loss to give metal sulfide (MS) indicating that the complexes will be good single source precursors for MS semiconductor nanoparticles. The potential of the complexes as single source precursors for semiconductor nanoparticles is being investigated.

## Supplementary Material

CCDC 762809 contains the supplementary crystallographic data for this paper. These data can be obtained free of charge via http://www.ccdc.cam.ac.uk/conts/retrieving.html, or from the Cambridge Crystallographic Data Centre, 12 Union Road, Cambridge, CB2 1EZ, UK; Fax: (+44)-1223-336-033 or E-Mail: deposit@ccdc.cam.ac.uk.

## Figures and Tables

**Table 1.** Temperature ranges of thermal decomposition and modes of various decomposition reactions.

**Complex**	**Decomposition temperature**	**DTG max. value**	**Decomposition reaction**	**Mass changes expet. found**	

Zn(S_2_CNmeC_6_H_5_)_2_	219–375	310	Zn(S_2_CNmeC_6_H_5_)_2_→ZnS ZnS→ZnO	3.05	3.60
	450–683			2.61	2.67

Cd(S_2_CNmeC_6_H_5_)_2_	270–372	324	Cd(S_2_CNmeC_6_H_5_)_2_→CdS CdS→CdO	2.92	3.25
	430–640			2.59	2.65

Hg(S_2_CNmeC_6_H_5_)_2_	168–361	299	Hg(S_2_CNmeC_6_H_5_)_2_→HgS Volatilization	4.16	2.50
	361–585			-	-

**Table 2.** Summary of crystal data and structure refinement for [(C_6_H_5_)(CH_3_)NCS_2_]_4_Hg_2_.

Compound	[(C_6_H_5_)(CH_3_)NCS_2_]_4_Hg_2_
Empirical formula	C_32_H_32_Hg_2_N_4_S_8_
Formula weight	1130.28
Temperature	100(2) K
Wavelength	1.54178
Crystal system	Monoclinic
Space group	P2_1/C_
Unit cell dimensions	
a (Å)	12.7168(10)
b (Å)	6.5198(6)
c (Å)	22.2612(19)
β (°)	98.341(3)
γ (°)	90
Volume (A^3^)	1826.2(3)
Z	2
D_calc_ Mg/m^3^	2.056 Mg/m^3^
Absorption coefficient (mm^−1^)	19.379
F(000)	1080
Crystal size (mm)	0.27 × 0.18 ×0.17
Theta range (°)	3.51 to 69.82
Limiting indices	−15 ≤ h ≤ 15, −7 ≤ k ≤ 7, −27 < +1 ≤ 26
Reflections collected	27791
Independent reflection	3412 [R(int) =0.0343]
Refinement method	Full-matrix least-squares on F^2^
Completeness to θ = 67.00	99.8 %
Data/restraints/parameters/	3412/0/210
Goodness-of-fit on F^2^	1.022
Final R indices [I > 2sigma(I)]	R1 = 0.0262, wR2 = 0.0716
R indices (all data)	R1 = 0.0265, wR2 = 0.0718
Largest diff. Peak and hole e. Å^−3^	1.836 and −1.181

**Table 3.** Selected Bond length (Å) and Bond Angle (°) for [(C_6_H_5_)(CH_3_)NCS_2_]_4_Hg_2_.

**Bond length (Å)**		**Bond Angle (°)**	
Hg(1)—S(3)	2.4114(9)	S(3)—Hg(1)—S(1)	146.42(3)
Hg(1)—S(1)	2.4810(9)	S(3)—Hg(1)—S(4)#1	102.35(3)
Hg(1)—S(4)#1	2.6955(9)	S(1)—Hg(1)—S(4)#1	107.67(3)
Hg(1)—S(2)	2.7327(9)	S(3)—Hg(1)—S(2)	122.15(3)
S(1)—C(1)	1.7404(4)	S(1)—Hg(1)—S(1)	69.91(3)
S(3)—C(9)	1.702(4)	S(4)#1—Hg(1)—S(2)	95.46(3)
S(4)—C(9)	1.732(4)	C(1)—S(1)—Hg(1)	88.17(13)
S(4)—Hg(1)#1	1.716(4)	C(1)—S(2)—Hg(1)	80.98(13)
N(1)—C(1)	1.347(5)	C(9)—S(3)—Hg(1)	99.34(13)
N(1)—C(3)	1.444(5)	C(9)—S(4)—Hg(1)#1	95.89(13)
N(1)—C(2)	1.469(5)	S(2)—C(1)—S(1)	120.9(2)
N(2)—C(9)	1.332(5)	N(1)—C(1)—S(2)	121.7(3)
N(2)—C(11)	1.454(5)	N(1)—C(1)—S(1)	117.4(3)
N(2)—C(10)	1.462(5)	N(2)—C(9)—S(4)	122.0(3)
		N(2)—C(9)—S(3)	116.2(3)
		S(4)—C(9)—S(3)	121.8(2)

Symmetry transformations used to generate equivalent atoms: #1, −x+2, −y, −z+1.
